# Hospitalization and Mortality in Brazilian Children and Adolescents Due to COVID-19: Retrospective Study

**DOI:** 10.2196/67546

**Published:** 2025-05-20

**Authors:** Ana Carolina Pereira de Godoy, Reinaldo Bulgarelli Bestetti

**Affiliations:** 1 Hospital da Criança e Materinade de Sao José do Rio Preto-Brazil, Rua Floriano Peixoto 2950, Sao José do Rio Preto, 15020010, Brazil, 55 11 949830105; 2 Faculdade de Medicina de Sao José do Rio Preto-Brazi, University of Ribeirão Preto, Sao José do Rio Preto, Brazil

**Keywords:** COVID-19, children, mortality, Brazil, retrospective study, morbidity, hospitalization

## Abstract

**Background:**

COVID-19 is currently one of the most important medical challenges as it affects the entire population, with children and adolescents being infected as easily as adults.

**Objective:**

The objective of this study was to evaluate the prevalence of mortality in children and adolescents aged <19 years, compared to that of adults.

**Methods:**

This retrospective, observational study analyzed the medical records of all patients diagnosed with COVID-19 by real-time reverse transcription–quantitative polymerase chain reaction who were hospitalized at Hospital de Base and the Infant and Maternal Hospital of São José do Rio Preto, São Paulo, Brazil. Out of a total of 8986 hospitalized patients who were COVID-19 positive, 383 (4.26%) were children and adolescents aged <19 years (group 1), and 8603 (95.74%) were adults (group 2).

**Results:**

Overall, mortality was significantly higher (*P*<.001) in group 2 (2185/8603, 25.4%) compared to group 1 (12/383, 3.1%). A total of 11 (92%) of the 12 patients in group 1 that died had associated diseases. The mortality rates by age group were as follows: infants aged <1 year, 1.6% (2/123); children aged 1-4 years, 4% (4/95); children aged 5-9 years, 2% (1/47); adolescents aged 10-14 years, 2% (1/40); and adolescents aged 15-19 years, 5% (4/78).

**Conclusions:**

Mortality from COVID-19 in children and adolescents was significantly lower than that in adults and was associated with other comorbidities.

## Introduction

COVID-19 is currently one of the most important medical challenges as it affects the entire population, with children and adolescents being infected as easily as adults; children and adolescents often remain asymptomatic or have mild complaints due to their immature immune systems [1]. Advanced age and comorbidities such as hypertension, diabetes, ischemic heart disease, vascular disease, renal failure, obesity, dyspnea, and dementia have been reported to be associated with a greater risk of death among older people infected with COVID-19. Some studies have described pathophysiological insights into COVID-19–induced coagulopathy, endothelium disease, and angiogenesis-associated defects [2-4]. Most children and adolescents with COVID-19 have mild symptoms; however, coagulopathies have been associated with multisystem inflammatory syndrome—a postinfectious complication—in young patients [2].

A study in Sergipe, Brazil, describing COVID-19 cases and deaths in children and adolescents reported 37 deaths before September 20, 2020, corresponding to a rate of 4.87 deaths per 100,000 of the population aged <19 years. Most children and adolescents had comorbidities such as chronic neurological diseases (n=7, 19%) and prematurity (n=4, 11%). Furthermore, most children and adolescents who died (n=18, 49%) were not admitted to intensive care units. This was possibly related to the great regional inequalities in health care [5].

A study in England reported that 4% (1408/35,200) of tests performed in children and adolescents aged <16 years were positive for SARS-CoV-2, compared to 19.1% to 34.9% in adults [3]. A review of pregnant women with COVID-19 reported that SARS-CoV-2 was detected in 4.3% (19/444) of newborns at birth. Elevated levels of immunoglobulin M and G serum antibodies were reported in one case, but the swab test was negative [4]. Mortality in children is higher in those with existing diseases compared to those without comorbidities [6].

Regarding the prognosis, metabolic acidosis, hyperlactatemia, hyperglycemia, altered liver function parameters, and hypoproteinemia are biochemical markers associated with the severity of the disease in children infected with SARS-CoV-2. Anemia and ketoacidosis are important risk factors for death in the pediatric patient population who are infected [7]. Gastrointestinal involvement is common in children hospitalized for acute COVID-19 infection and multisystem inflammatory syndrome, but gastrointestinal involvement is not associated with critical illness, length of hospital stay, or mortality in acute cases of COVID-19 [8].

In a study of pediatric COVID-19 epidemiology, Sousa et al [9] highlighted the presence of comorbidities in patients aged <2 years, in particular those related to congenital heart disease, Down syndrome, obesity, and asthma. Furthermore, these authors reported differences in morbidity and mortality due to regional disparities, with higher rates in regions with less resources, as is the case in the economically less favored northern and northeastern regions of Brazil. In their analysis of 2020 deaths of children that occurred throughout Brazil, 42% occurred in children aged <2 years and 43% occurred in adolescents aged 10‐19 years, with children aged 2‐10 years being relatively protected. A total of 58% of deceased patients had at least one comorbidity. It is noteworthy that 69% of deaths were observed in Black or multiracial patients, 25.5% in White patients, and 5% in Indigenous patients, with approximately 60% occurring in the northern and northeastern regions of Brazil.

It is also worth remembering the prolonged symptoms that are associated with COVID-19—notably, fatigue, headaches, drowsiness, and difficulty concentrating—as well as the impact of social isolation, which can cause psychosocial disorders and learning gaps that can have a major impact on children’s education [10,11].

An American study reports that pediatric patients with a recent relapse of cancer have a higher chance of all-cause mortality when infected with COVID-19 [12].

The objectives of this study were to evaluate the mortality of children and adolescents and compare it with that of adults in a cohort of 8986 patients hospitalized for COVID-19 in a university hospital complex in Brazil.

## Methods

### Patients and Setting

A retrospective, observational study was performed to analyze the medical records of all patients diagnosed with COVID-19 who were hospitalized at Hospital de Base and the Infant and Maternal Hospital of São José do Rio Preto, São Paulo, Brazil, from March 2020 to July 2023.

All patients diagnosed with COVID-19 by reverse transcription–quantitative polymerase chain reaction and admitted to these hospitals were included in this study. Patients hospitalized for other illnesses and those who tested negative for COVID-19 during this period were excluded.

Patients aged <19 years were categorized as children or adolescents (group 1), and those aged ≥19 years were categorized as adults (group 2). Group 1 was further subdivided by age: <1 year, 1‐4 years, 5‐9 years, 10‐14 years, and 15‐19 years. The monthly occurrence of COVID-19 was evaluated, with mortality in children and adolescents being compared to that in adults. Existing diseases and the occurrence of other viral infections were identified over the same period in patients aged <19 years who were COVID-19 positive.

### Ethical Considerations

The study was approved by the ethics committee of the Medical School in São José do Rio Preto under approval 6.086.468 (CAAE 67915723.3.0000.5415; date approved: May 29, 2023).

For this study, the institution permitted the analysis of these records, guaranteeing the use of data only in this research protocol. Absolute confidentiality was maintained during the data collection and use thereof. Safeguards were taken to collect data from medical records in the institution’s own archives department; patient charts were not removed for any reason.

The signed consent form was authorized by the ethics committee to be waived, because these were medical records and due to the impossibility of contacting family members and those who have died. Thus, the authors guaranteed the privacy and confidentiality of the data obtained, fully preserving the anonymity of the participants in accordance with the Declaration of Helsinki.

### Statistical Analysis

Data were tabulated in Microsoft Excel spreadsheets, with statistical analysis conducted using StatsDirect 3 software (StatsDirect Ltd). Descriptive statistics, Fisher exact test, chi-square test, and odds ratio were used considering an α error of 5% (*P*≤.05).

## Results

**Figure 1.**
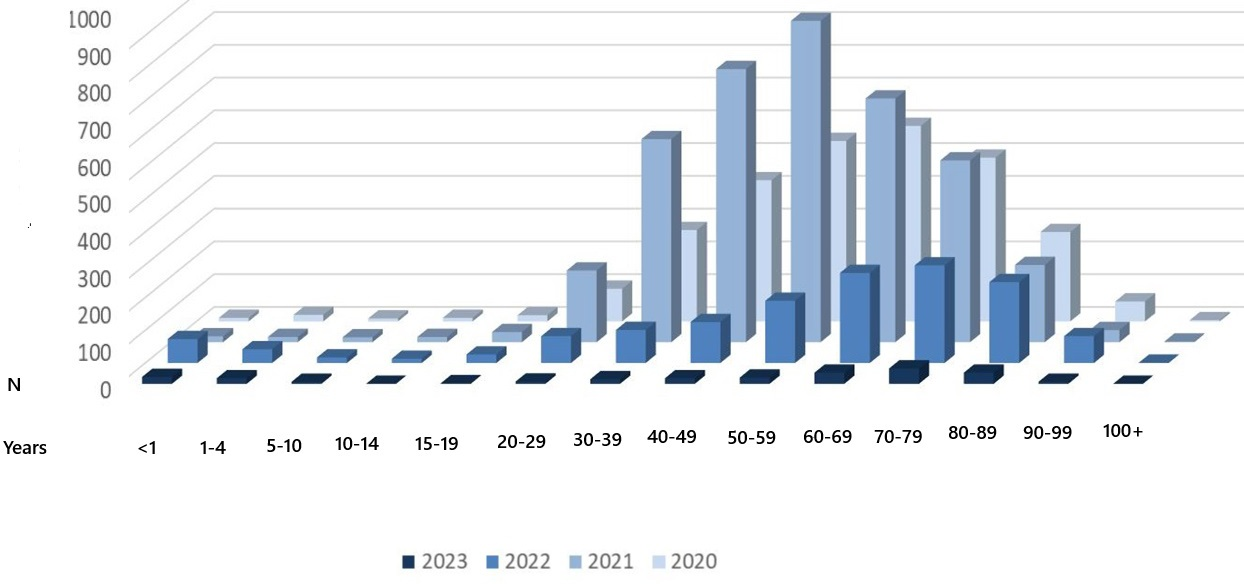
Patients hospitalized with COVID-19 by age group and year.

Between March 2020 and July 2023, a total of 8986 patients who were COVID-19 positive were hospitalized, 383 (4.26%) of whom were aged <19 years and 8986 (95.74%) were aged ≥19 years (mean age 57.66 y; Figure 1). Overall, 12 children and adolescents died, that is, 3.1% of the 383 patients in this age group. The number of deaths in group 2 (adults) was 2185, that is, equivalent to 25.4% of the 8603 hospitalized adults and 99.45% (2185/2197) of overall deaths. Mortality was significantly higher for adults than children and adolescents aged <19 years (Yates-corrected *χ*^2^ test=98.127501, *P*<.001; odds ratio 10.317, 95% CI 5.793‐18.374).

**Figure 2.**
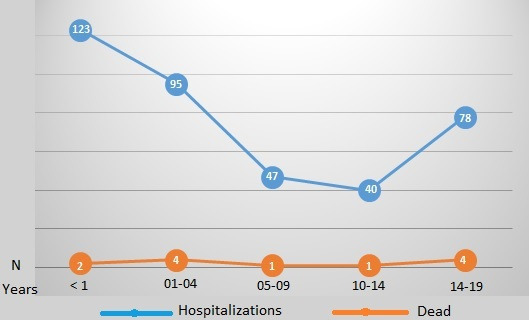
Hospitalizations and deaths of children and adolescents who were COVID-19 positive.

**Figure 3.**
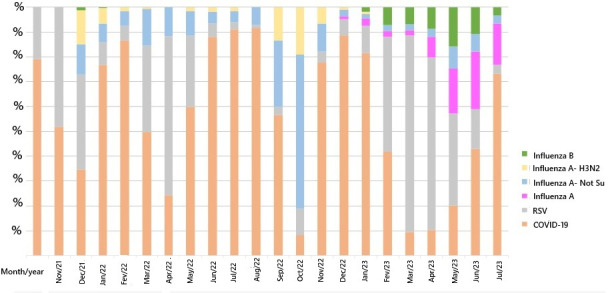
Percentage of hospitalized cases for COVID-19, influenza, and respiratory syncytial virus (RSV) at the hospital complex in São José do Rio Preto, according to month and etiological agent from October 1, 2021, to July 24, 2023.

Regarding the different age groups of children and adolescents, the mortality rates were as follows: infants aged <1 years, 1.6% (2/123); children aged 1-4 years, 4% (4/95); children aged 5-9 years, 2% (1/47); adolescents aged 10-14 years, 2% (1/40); and adolescents aged 15-19 years, 5% (4/78). There were no statistical differences between the different age groups (Fisher exact test: *P*=.30; Table 1 and Figure 2). Figure 3 shows the main viral infections of the patients who were COVID-19 positive in the 2 hospitals, emphasizing the importance of this disease. Table 2 shows the associated diseases of the 12 children who died from COVID-19.

**Table 1.** Hospitalizations and deaths for different age groups from March 2020 to July 2023.

Age group (years)	2020		2021		2022		2023		Total	
	Hospitalizations, n	Deaths, n	Hospitalizations, n	Deaths, n	Hospitalizations, n	Deaths, n	Hospitalizations, n	Deaths, n	Hospitalizations, n	Deaths, n
<1	11	0	18	1	73	1	21	0	123	2
1‐4	20	1	16	1	42	2	17	0	95	4
5‐9	9	0	15	1	17	0	6	0	47	1
10‐14	11	1	16	0	13	0	0	0	40	1
15‐19	19	2	31	0	26	2	2	0	78	4
20‐29	100	8	219	20	82	4	7	1	408	33
30‐39	279	13	620	76	101	8	14	2	1014	99
40‐49	431	27	834	125	125	11	17	3	1407	166
50‐59	551	85	981	241	190	23	19	3	1741	352
60‐69	597	147	744	287	275	71	34	11	1650	516
70‐79	500	187	555	257	298	90	47	11	1400	545
80‐89	273	141	236	136	247	94	34	6	790	377
90‐99	61	43	37	22	82	28	6	0	186	93
>100	4	3	2	1	1	0	0	0	7	4
Total	2866	658	4324	1168	1572	334	224	37	8986	2197

**Table 2.** Chief comorbidities associated with COVID-19 that led to the deaths of children and adolescents.

Age (y)	Sex	Comorbidity	Infection	Antibiotics	Image	Vasoactive drugs	Mechanical ventilation	Intensive care unit	Dialysis support	Time in hospital (mo)
19	Female	Hodgkin lymphoma	Yes	Yes	CT^a^ 25% compromised (ground-glass opacity)	Yes	Yes	Yes	Yes	45
14	Female	Down syndrome	Yes	Yes	CT 70%	Yes	Yes	Yes	Yes	12
2	Male	Down syndrome and heart disease	Yes	Yes	Rx^b^	Yes	Yes	Yes	Yes	5
19	Female	Down syndrome, schizophrenia, asthma, and corrected heart disease	Yes	Yes	CT 50% compromised	Yes	Yes	Yes	Yes	5
0	Male	None	Yes	Yes	Rx infiltrated	Yes	Yes	Yes	Yes	5
2	Male	Encephalopathy	Yes	Yes	Rx	Yes	Yes	Yes	Yes	24
5	Male	Renal transplant	Yes	Yes	CT >50% infiltrated (ground-glass opacity)	Yes	Yes	Yes	No	20
3	Male	Congenital heart disease	Yes	Yes	Rx	Yes	Yes	Yes	No	3
16	Female	Tumor in the central nervous system	Yes	Yes	CT >50% infiltrated (ground-glass opacity)	Yes	Yes	Yes	Yes	10
17	Female	Lupus	Yes	Yes	CT <25%	Yes	Yes	Yes	No	55
3	Female	Hydranencephaly	Yes	Yes	Rx	No	Yes	Yes	Yes	1
1	Female	Congenital heart disease	No	No	Rx	Yes	Yes	No	No	1

a CT: computed tomography.

b Rx: x-ray.

## Discussion

### Principal Findings

This study reports on the general mortality of children and adolescents aged <19 years and adult patients in a single university hospital complex from March 2020 to July 2023. Of the 8986 patients hospitalized with COVID-19, a total of 383 (4.26%) were children and adolescents treated at the children’s hospital. Deaths in this age group represented 0.55% (12/2197) of all deaths from COVID-19 in the institution and 3.1% (12/383) of all hospitalized patients aged <19 years. No significant difference was detected in mortality between the age groups of children and adolescents. A multicenter study in Latin America reported a mortality rate of 14% in 210 children, with 67% of those who died being treated in an intensive care unit [13].

Observational studies in the pediatric population have shown that the presence of comorbidities is a risk factor for severe disease. Obesity, genetic disorders (such as sickle cell anemia), neurological disorders, hematological diseases, congenital heart diseases, diabetes, chronic kidney disease, asthma, and other lung diseases are pathologies that have already been associated with the severity of COVID-19 in previous studies [14]. In this study, heart disease was the main disorder reported in the children and adolescents who died.

Regarding the age of the children and adolescents, there is no consensus on which age group has the highest or lowest severity of disease, included mortality; however, severity does seem to be linked to the presence of preexisting clinical factors. A review study reported that younger children and those with specific comorbidities, such as obesity, diabetes, heart diseases, chronic lung diseases, epilepsy, and immunocompromised conditions, are at higher risk of infection and potentially more severe consequences of COVID-19 [15,16].

One interesting Brazilian study that analyzed a large nationwide database of hospitalized children and adolescents with laboratory-confirmed COVID-19 showed that death was associated with being aged younger than 2 years or between 12 and 19 years. Indigenous ethnicity, living in the poorest microregions, and the presence of comorbidities were also correlated with the severity of the disease. Therefore, health care disparities and social inequalities, exacerbated by interweaving comorbidities, might have contributed synergistically to magnifying the COVID-19 burden for more socioeconomically deprived and vulnerable individuals [17]. Due to uncertainties in epidemiological data on children, there is still much to learn about the manifestations of COVID-19 in this population.

In this study, no significant differences with respect to mortality were detected between age groups of the children and adolescents; however, the number of patients is too small to arrive at any definite conclusion.

In North America, mortality was higher in children younger than 1 year, followed by adolescents aged between 15 and 19 years [18]. In 2021 and 2022, a study in Java, Indonesia, analyzed 6441 patients aged ≤18 years who were positive for COVID-19 and reported that 2.7% of deaths occurred with other associated factors; in this study, the rate was 91.67%—a difference that may be related to the different conditions (socioeconomic factors and access to health care) in the 2 countries [19].

Regarding mortality, since the beginning of the pandemic, newborns, children, and adolescents are less prone to this emerging condition compared to adults. Most of them experience mild symptoms; hospitalization and death of pediatric patients are rare, with deaths usually being explained by associated complications [20].

In Ecuador, a study on mortality from 2020 to 2021 reported that out of 34,001 confirmed cases of COVID-19, a total of 258 were children and adolescents aged between 0 and 19 years and that 127 died due to COVID-19. In the same period, the study found that most deaths occurred in children aged 0 to 1 year, representing 44% (n=114) of the total deaths reported [21].

In Brazil, in 2020, a total of 14,638 children were diagnosed with SARS, resulting in 1180 (8.06%) deaths. Being younger than 2 years was a risk factor for higher hospitalization and mortality rates [22]. In 2021, an increase in the number of cases of pediatric patients was noted; according to data from the Brazilian Ministry of Health, there were 17,644 occurrences of SARS-CoV-2 with 1263 (7.15%) deaths. This rise is related to greater knowledge of the disease, a simpler diagnosis, the larger quantity of diagnostic tests performed, and the emergence of new variants (Delta and Omicron) [9].

Pediatric patients with comorbidities are at higher risk for hospitalization and mortality. Among the reported comorbidities, the following should be highlighted: Down syndrome, asthma, obesity, immunosuppression, and heart disease. Morbidity and mortality due to COVID-19 are not similar to other etiologies of acute respiratory distress syndrome. This is because fulminant activation of coagulation cascades can occur in COVID-19, resulting in widespread microvascular thrombosis and the consumption of clotting factors [9,22-25]. It appears that inflamed lung tissues and pulmonary endothelial cell damage may result in the formation of microthrombi that contribute to the high incidence of thrombotic complications, such as deep vein thrombosis, pulmonary embolism, and arterial thrombotic complications.

Another important factor that must be highlighted is the possibility of long-term symptoms such as fatigue, headaches, drowsiness, and difficulty concentrating, all of which have implications in social and educational spheres, leading to important concerns regarding the impact of the pandemic on future education levels.

Another point to be considered is related to the risk of the long-term effects of COVID-19 on the health of the general population. Post–COVID-19 coronary changes and the presence of multisystem inflammatory syndrome were identified in some pediatric patients. Therefore, although children and adolescents represent a low percentage of COVID-19 cases in Latin America and worldwide, major social, economic, and cultural implications are linked to the disease in this population. In truth, COVID-19 has disrupted all spheres of life, including country risk factors, such as exposure to multidimensional risk drivers.

Socioeconomic and political factors significantly influence health care conditions and directly impact the response to pandemics, including the allocation of resources for immediate health care needs. Studies analyzing the influence of demographic, economic, and political conditions in different countries and continents show a significant difference between countries with a low Human Development Index and those with a high Human Development Index. This has influenced, and continues to directly influence, human health care [26,27].

It should be remembered that the dynamics of infections and deaths related to COVID-19 differ from country to country and are constantly changing throughout Latin America. Management requires political leadership, financial resources, and social equality, as well as the existence of informal and regional economies directed toward disadvantaged populations that traditionally have had limited access to health services. In this study, COVID-19 was the most common viral infection identified in this period. Mortality from COVID-19 in children and adolescents was lower than that in adults and was generally associated with existing diseases, with the main one being heart disease.

This study shows results consistent with our hypothesis and with the results of studies published in MEDLINE. Thus, predicting the outcome of COVID-19 is of vital clinical importance to better allocate medical resources and provide individualized treatment for patients. The availability of clinical characteristics and parameters with potential prognostic implications will be of value for effective prevention and intervention.

### Conclusion

Mortality from COVID-19 in children and adolescents was lower than that in adults. A second interesting finding was that these deaths were generally associated with existing diseases.
